# Periostin: a potential biomarker for diagnosis and treatment monitoring in canine atopic dermatitis

**DOI:** 10.1080/01652176.2025.2564447

**Published:** 2025-09-26

**Authors:** Keon Kim, Chang-Yun Je, In Su Seo, Yoon Jung Do, Woong-Bin Ro, Chang-Min Lee

**Affiliations:** a Department of Veterinary Internal Medicine, College of Veterinary Medicine and BK 21 FOUR program, Chonnam National University, Gwangju, Republic of Korea; b Laboratory Animal Medicine, College of Veterinary Medicine, Chonnam National University, Gwangju, Republic of Korea; c Division of Animal Diseases & Health, National Institute of Animal Science, Rural Development Administration, Wanju-gun, Republic of Korea

**Keywords:** Atopic dermatitis, biomarker, canine, periostin, T-helper 2

## Abstract

Canine atopic dermatitis (cAD) is a chronic inflammatory skin condition associated with T helper 2 (Th2)-type immune responses, and recent evidence suggests that periostin, an extracellular matrix protein, may play a role in its pathogenesis. To investigate the significance of serum periostin concentrations in dogs with cAD, this study examined their relationship with disease severity and laboratory parameters, including eosinophil counts and Th2 cytokines such as interleukin (IL)-4 and IL-13. A total of 68 dogs diagnosed with cAD and 21 healthy controls were included, with serum periostin, IL-4, and IL-13 levels measured. Disease severity was assessed using the Canine Atopic Dermatitis Extent and Severity Index (CADESI-04) and the Pruritus Visual Analog Scale (PVAS). The results showed that serum periostin concentrations were significantly higher in dogs with cAD compared to healthy controls and exhibited a positive correlation with CADESI-04 scores, reflecting disease severity. Treatment with prednisolone or oclacitinib led to a significant reduction in serum periostin, IL-4, and IL-13 levels, along with a decrease in eosinophil counts. These findings highlight the potential of serum periostin as a valuable biomarker for assessing cAD severity and monitoring treatment response, emphasizing its clinical relevance as an objective measure.

## Introduction

Canine atopic dermatitis (cAD) is described as an allergic skin condition that is both inflammatory and pruritic and is commonly linked to genetic factors. This disease typically involves immunoglobulin E (IgE) antibodies reacting to environmental allergens and is associated with distinct clinical signs (Halliwell 2006; Chaudhary et al. 2019). The pathogenesis of cAD has not yet been fully elucidated; however, it shares many similarities with human atopic dermatitis (AD) including IgE-mediated hypersensitivity and defects in the epidermal barrier (Lian and Halliwell 1998; Marsella and Samuelson 2009). Meanwhile, recent studies have suggested that, unlike in human AD, the evidence supporting a primary skin barrier defect as a pathogenic factor in cAD is relatively weak (Combarros et al. 2025). Moreover, cAD also exhibits features of allergic inflammation accompanied by T helper 2 (Th2)-type immune response (Olivry et al. 1999; Nuttall et al. 2002). This is supported by previous studies showing that Th2 cytokines such as interleukin (IL)-4 and IL-13 are found in higher concentrations in the lesions of atopic skin and in the serum of dogs with cAD than in those of healthy dogs (Hayashiya et al. 2002; Schlotter et al. 2011; Majewska et al. 2016). A study also reported an increased presence of IL-4-expressing helper T cells in the peripheral blood of dogs affected by cAD (Martins et al. 2018). Consequently, cAD is an inflammatory disease associated with Th2 responses, akin to human AD. To manage the clinical signs of cAD, immunomodulatory and anti-pruritic treatments such as prednisolone (a glucocorticoid) and oclacitinib (a Janus kinase inhibitor) are commonly used to suppress Th2-associated cytokines, thereby effectively alleviating inflammation and pruritus (Olivry et al. 2015).

Periostin, a member of the fasciclin family, is an extracellular matrix protein that regulates cellular functions by binding to integrin molecules on the cell surface (Morra and Moch 2011; Yamaguchi 2014). It plays a crucial role in providing signals for tissue development and remodeling (Takeshita et al. 1993; Norris et al. 2009). Furthermore, expression of periostin is markedly upregulated in response to Th2-associated cytokines including IL-4 and IL-13, which are predominantly expressed in allergic diseases such as asthma and AD (Zhou et al. 2010; Masuoka et al. 2012; Pulendran and Artis 2012). Recent research has advanced the understanding of the role of periostin in the development of AD. A periostin knock-out mouse model demonstrated that periostin is essential for the manifestation of allergic skin inflammation. Periostin acts as an intrinsic mediator that plays a pivotal role in amplifying and sustaining allergic skin inflammation by driving Th2-type immune responses and activating keratinocytes (Masuoka et al. 2012; Taniguchi et al. 2014). In human medicine, it has been discovered that serum periostin concentrations play a role in the pathogenesis of AD in both adults and children (Kou et al. 2014; Sung et al. 2017).

Several studies have reported that the periostin gene is highly expressed in the skin lesions of cAD, similar to observations in human AD (Mineshige et al. 2015; Mineshige et al. 2018). However, there has been no research on the evaluation of serum periostin concentrations in canine AD. In this study, the authors aim to investigate the relationship between serum periostin concentrations and disease severity in cAD, and to evaluate the potential of periostin as an objective biomarker for disease assessment and monitoring of treatment response.

## Materials and methods

### Serum sampling

Serum periostin concentrations were assessed using serum samples obtained from dogs with cAD as well as healthy dogs visiting the clinic for routine vaccinations or examinations presented at the Chonnam national university veterinary teaching hospital and local animal hospitals in Republic of Korea. All blood samples were collected at the time of first presentation. Serum was separated by centrifugation at 4,000 RPM for 5 min within 1 h of collection and stored at −20 °C for less than one year until analysis. Approval for the experimental design was granted by the institutional animal care and use committee of the university under approval number CNU IACUC-YB-2024-61.

### Animals

The group allocation was as follows: 68 dogs were included in the cAD group, among which 20 dogs were further classified into the remission group based on the CADESI-04 system. Meanwhile, the cAD treatment group consisted of 20 dogs, of which 3 had been previously included in the cAD group.

The cAD group (*n* = 68) was retrospectively selected from dogs with cAD at their initial presentation to a veterinary teaching hospital and local animal hospital in 2024. The diagnosis of cAD was based on a compatible history and clinical examination with the exclusion of other differential diagnoses such as hormonal diseases, bacterial dermatitis, *Malassezia* spp. dermatitis, flea allergy dermatitis, and demodicosis or scabies and the inclusion of at least five of Favrot’s criteria set II. Food allergies were excluded based on an incomplete or no response to an 8-week elimination diet using hydrolyzed protein-based commercial food (Favrot et al. 2010; Hensel et al. 2015).

At the first presentation, except for cAD remission group, all cAD dogs were excluded if the following treatment washout periods had not been confirmed by history: anti-inflammatory or antipruritic drugs within 2 weeks; corticosteroid medication by oral or topic route within 4 weeks. Most dogs diagnosed with cAD underwent assessment of the Canine Atopic Dermatitis Extent and Severity Index-04 (CADESI-04) score and the pruritus visual analog scale (PVAS) score. The CADESI-04 score was evaluated by at least two veterinarians, meaning that the investigators were not always the same individuals. The PVAS score was recorded based on a formal visual analog scale, which was presented to the owner during the visit and marked directly by the owner. According to the previous study (Olivry et al. 2014), clinicians evaluated three types of skin lesions—erythema, lichenification, and excoriation/alopecia—using a four-point severity scale (0 = none, 1 = mild, 2 = moderate, 3 = severe). Assessments were performed across 20 different body regions, with three lesion types and four severity grades considered, resulting in a maximum possible score of 20 × 3 × 3 = 180. Based on the CADESI-04 scoring system, cutoff values for disease severity were defined as follows: remission <10, mild 10–34, moderate 35–59, and severe ≥60. Among the dogs diagnosed with cAD in the study, several received continuous treatment following their first presentation to the veterinary hospitals, and oral prednisolone (PDS) or oclacitinib was prescribed as needed in certain cases.

The cAD remission group (*n* = 20) was composed of dogs with a history of cAD diagnosis whose clinical signs had either resolved or substantially decreased due to interventions such as medication treatment or environmental adjustments, as determined at the time of initial presentation in our participating veterinary hospitals. These individuals exhibited the CADESI-04 score of less than 10 and PVAS score of less than 2. In this study, the remission group consisted of dogs with cAD that met the following criteria: (1) first-time presentation to one of the veterinary hospitals participating in the study; (2) fulfillment of the remission criteria, including CADESI-04 and PVAS scores; and (3) a clear history of treatment or management for cAD at another veterinary hospital.

The healthy control group (*n* = 21) was composed of clinically normal dogs showing no abnormalities on physical and dermatological examinations. All control dogs were client-owned dogs. Both the cAD and control groups excluded dogs with cardiovascular, skeletal, neoplastic, or systemic inflammatory diseases that could potentially elevate periostin concentrations as well as those younger than one year of age (Bonnet et al. 2016; Landry et al. 2018; Izuhara et al. 2019).

The cAD treatment group (*n* = 20) was retrospectively analyzed using serum samples from dogs diagnosed with cAD that were available for follow-up after receiving additional treatment, including either PDS or oclacitinib. These dogs were diagnosed under the same criteria as the cAD group, and serum samples were re-evaluated within two weeks of medication. Specifically, PDS was prescribed to a total of 5 dogs at a dosage of 0.5 mg/kg BID PO for one week, while oclacitinib was prescribed to a total of 15 dogs at a dosage of 0.4 mg/kg BID PO for two weeks. Of the total 20 dogs in the cAD treatment group, only 3 dogs with a clear history of washout periods were re-included in the cAD group for further analyses. The remaining 17 dogs were excluded due to unclear washout histories.

### Measurement of serum biomarkers and eosinophil counts

#### Measurements of serum periostin concentrations

Anti-canine periostin monoclonal antibody was pre-coated on ELISA plates; then, *via* washing, all antibodies and impurities that did not bind to the plate were removed (Cat No. MBS2606982, MyBioSource, San Diego, CA, USA). One hundred microliters of sera diluted twenty-fold with sample diluent was added and incubated at 37 °C for 90 min. The target analyte in the sample binds to the capture antibodies, forming an antigen-antibody complex. After washing, a biotin-labeled polyclonal antibody specific for the target analyte was added, forming an antibody-antigen-antibody complex. The plates were washed again, and an enzyme-conjugated solution that binds to the biotin-labeled antibodies was added. After further washing, a substrate solution was added, resulting in a color change due to the enzyme activity, which is correlated with the quantity of the target analyte in the sample. The reaction was terminated with a stop solution, and the absorbance was measured at 450 nm with a microplate reader (BioTek, Winooski, VT, USA). The test was performed twice within the same day. The test results were interpreted using a calibration curve, and the target analyte concentration was calculated based on this curve. The optical density measurements showed a proportional increase corresponding to the concentrations of the recombinant canine protein (R^2^ = 0.9939, *p <* 0.001).

#### ELISA for IL-4 and IL-13

To measure the concentrations of IL-4 and IL-13, specific canine ELISA kits (MyBioSource, San Diego, CA, USA) were used. Similar to the measurement of periostin, each assay was performed in accordance with the manufacturer’s instructions.

#### Eosinophil count measurement

A complete blood cell count analyzer (Procyte Dx Analyzer, IDEXX Veterinary Diagnostics, USA) was used to measure hematological parameters.

### Statistical analysis

Statistical analyses were performed using IBM SPSS Statistics (version 26, IBM Corp., USA) and GraphPad Prism (version 10.2, GraphPad Software Inc., USA). The Shapiro-Wilk test was applied to evaluate the normality of the data. The Mann-Whitney U-test was used to analyzed difference in serum periostin concentrations, IL-4 and IL-13 levels, and eosinophil counts between dogs with cAD and healthy controls. The Kruskal-Wallis test was used to compare among the control group and cAD group based on CADESI-04 scores (mild group and moderate-to-severe group). In this study, the moderate and severe groups were combined for statistical analysis. Pearson’s correlation analysis was performed to evaluate associations between serum periostin concentrations and the CADESI-04 score, PVAS score, eosinophil count, and IL-4 and IL-13 levels in cAD group followed by Shapiro-Wilk normality test. Pearson’s correlation analysis was conducted to confirm associations between serum periostin level and IL-13 in healthy control group, while Spearman’s correlation analysis was done to confirm between periostin and IL-4, eosinophil count. The Wilcoxon matched-pairs signed-rank test was used to compare serum periostin concentrations, IL-4 and IL-13 levels, and eosinophil counts between pre-treatment and post-treatment in dogs with cAD.

## Results

### Study population

Sixty-eight dogs diagnosed with cAD were included in this study. The ages of the animals ranged from 1 to 12 years, with a mean age of 7.5 ± 3.1 years. The group consisted of 40 females, 35 of which were spayed, and 28 males, 25 of which were neutered. Twelve breeds were represented, with Maltese and Poodles being the largest groups including 18 and 14 dogs, respectively (Table 1). The CADESI-04 score was available for 59 dogs based on their chart data. According to the CADESI-04 scores, 20 dogs were classified as in remission (score <10), 22 dogs as having mild disease (scores 10–34), 12 dogs as having moderate disease (scores 35–59), and 5 dogs as having severe disease (score ≥60). Moderate and severe group (*n* = 17) were grouped to make the statistics in this study. The PVAS was used in 61 dogs, with scores ranging from 0 to 10 (median, 2.25; interquartile range [IQR] 1.375–4.25).

**Table 1.** Signalments of all dogs included in the study.

Variables		cAD group *N* = 68	Control *N* = 21
Breed	Maltese	18	8
	Poodle	14	2
	Bichon Frise	7	2
	Shih Tzu	7	0
	French Bulldog	5	0
	Mixed	5	2
	Pomeranian	3	2
	Dachshund	3	0
	Golden Retriever	2	0
	Yorkshire Terrier	2	1
	Chihuahua	1	1
	Cocker Spaniel	1	0
	Beagle	0	1
	Schnauzer	0	1
	Poongsan	0	1
Sex	Intact Female	5	0
	Spayed Female	35	7
	Intact Male	3	2
	Neutered Male	25	12
Age (years)	Mean age ± SD	7.5 ± 3.1	7.3 ± 3.7

Abbreviation: SD, Standard deviation.

The control group consisted of 21 dogs, with ages ranging from 1 to 15 years, with a mean age of 7.3 ± 3.7 years. This group included 10 females (all spayed) and 14 males (12 neutered). The most represented breed was the Maltese (Table 1).

Among the samples from the cAD and healthy control groups in which serum periostin concentrations were quantified, Th2-related cytokines were additionally measured in samples with sufficient volume for further analysis. IL-4 was measured in total 74 dogs (cAD = 55, healthy = 19), and IL-13 was measured in total 71 dogs (cAD = 52, healthy = 19). In addition, eosinophil count data were obtained from total 56 dogs (cAD = 42, healthy = 14) with available CBC records through medical chart review.

### Comparison of serum periostin concentrations and other biomarkers in dogs with cAD and healthy dogs

**Figure 1.**
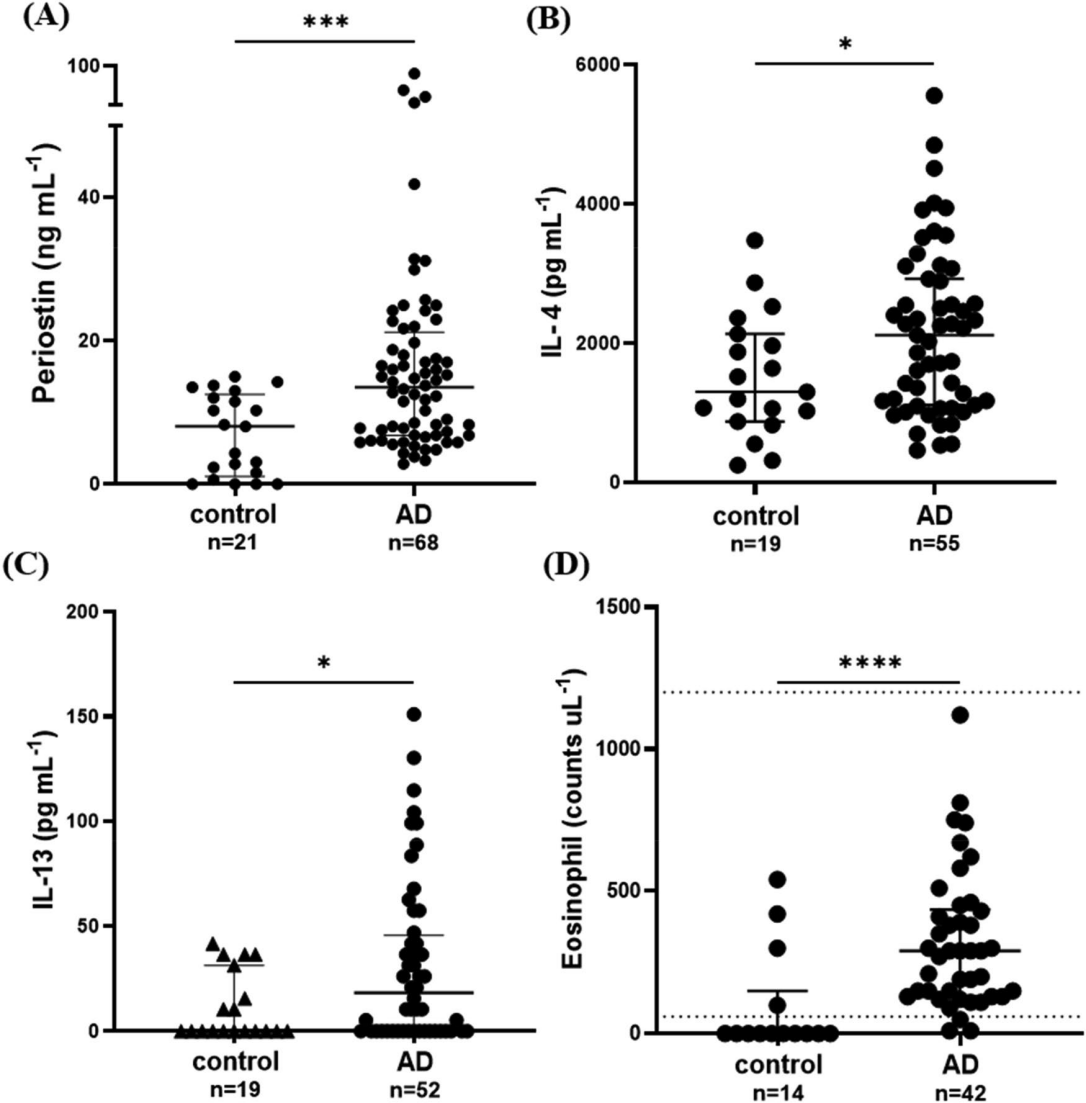
Comparison of the median periostin concentrations, IL-4 and IL-13 levels, and eosinophil counts between healthy controls and dogs with cAD. The scatter dot plot presents the median serum periostin concentration (A), IL-4 level (B), IL-13 level (C), and eosinophil count (D) across groups. The dotted line represents the normal reference range for eosinophil counts. Median values are displayed as a line, while the 25^th^ and 75^th^ percentiles are indicated as boundaries. Statistical analysis was performed using the Mann–Whitney U-test, with *p < 0.05 and ***p < 0.001 indicating significance.

Dogs with cAD exhibited significantly higher median serum periostin concentrations than healthy dogs (median, 13.51 ng/mL; IQR, 6.801–21.21 versus median, 8.044 ng/mL; IQR, 1.086–12.52; *p* < 0.001) (Figure 1A). Furthermore, dogs with cAD exhibited significantly higher median serum levels of IL-4 and IL-13 than healthy controls (IL-4: median 2,116 pg/mL, IQR 1,112–2,925 vs. median 1,300 pg/mL, IQR 874.9–2,132; IL-13: median 18.24 pg/mL, IQR 0–45.58 vs. median 0 pg/mL, IQR 0–31.26; both *p* < 0.05) (Figure 1B,C). Furthermore, the eosinophil counts were also elevated in dogs with cAD compared with healthy controls (median 290/µL, IQR 130.0–435.0 vs. median 0/µL, IQR 0–150.0; *p* < 0.0001) (Figure 1D).

### Comparison of serum periostin concentrations between groups according to the CADESI-04 score

The serum periostin concentrations in dogs with cAD in remission were higher than those of healthy controls (median: 10.40 ng/mL, IQR: 6.056–15.87 vs. median: 8.044 ng/mL, IQR: 1.086–12.52; *p* < 0.05).

**Figure 2.**
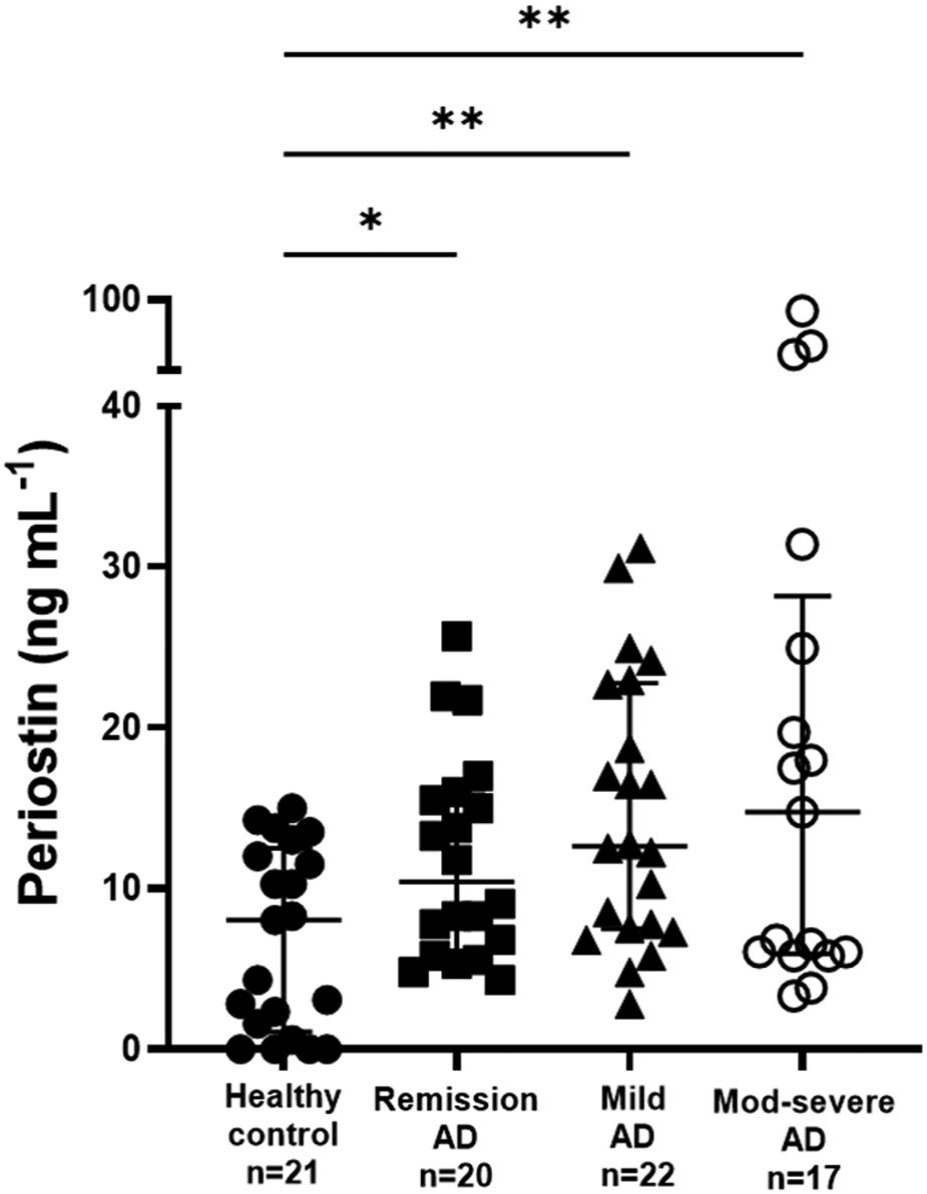
Comparison of serum periostin concentrations between groups according to the CADESI-04 score. The line represents the median value, while the boundaries indicate the 25^th^ and 75^th^ percentiles. Statistical significance was determined using the Kruskal–Wallis test, with **p < 0.01 and *p < 0.05.

Similarly, serum periostin concentrations were elevated in dogs with mild cAD, with a median of 12.64 ng/mL (IQR: 7.484–22.77). Furthermore, dogs with moderate to severe cAD had higher serum periostin concentrations than healthy controls (median: 14.75 ng/mL, IQR: 5.931–28.17 vs. median: 8.044 ng/mL, IQR: 1.086–12.52; *p* < 0.01). However, no statistically significant differences were observed in serum periostin concentrations between the remission group and both the mild cAD group and the moderate to severe cAD group (Figure 2).

### Correlation of serum periostin concentrations with clinical scores and other biomarkers of cAD

#### Correlation between serum periostin concentrations and CADESI-04 scores

**Figure 3.**
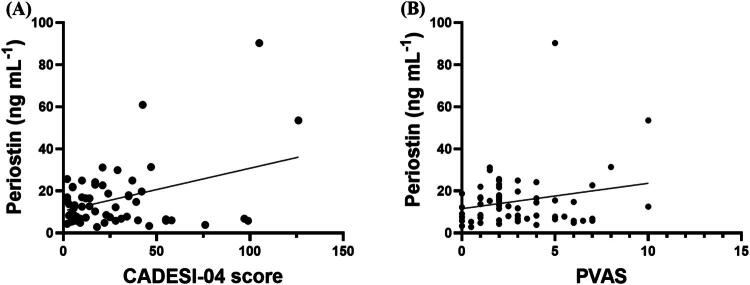
Correlation between serum periostin concentration and CADESI-04 and PVAS scores. Scatter plots showing serum periostin concentrations in relation to CADESI-04 (A, n = 59) and PVAS scores (B, n = 61).

A weak to moderate positive correlation was identified between serum periostin concentrations and CADESI-04 scores, with a Pearson correlation coefficient of *r* = 0.381 (*p* < 0.01) (Figure 3A).

#### Association between serum periostin concentrations and PVAS scores in dogs with cAD

Pearson’s correlation analysis indicated no significant association between serum periostin concentrations and PVAS scores (*r* = 0.2188, *p* = 0.09) (Figure 3B).

#### Correlation between periostin concentrations and other biomarkers in dogs with cAD

**Figure 4.**
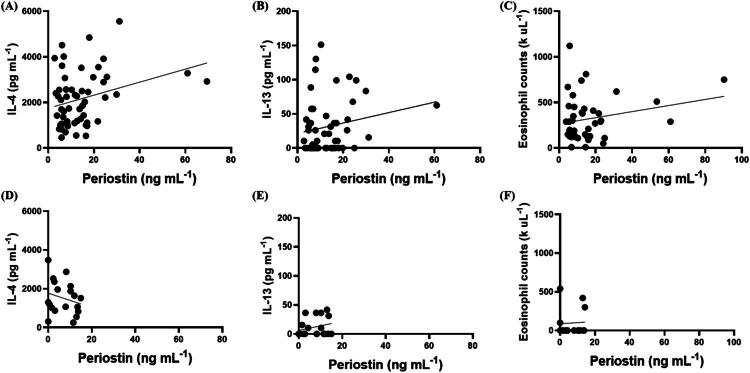
Correlation between serum periostin concentrations and IL-4 and IL-13 levels and eosinophil counts in cAD and control group. Scatter plots showing serum periostin concentrations in cAD group associated with IL-4 levels (A, n = 55), IL-13 levels (B, n = 52), and eosinophil counts (C, n = 42) and serum periostin in healthy control group associated with IL-4 levels (D, n = 19), IL-13 levels (E, n = 19), and eosinophil counts (F, n = 14). Pearson’s correlation analysis revealed a weak positive correlation between serum periostin concentrations and IL-4 in dogs with cAD (r = 0.295, *p* < 0.05).

Pearson’s correlation analysis for dogs with cAD showed a weak correlation between serum periostin concentrations and IL-4 levels (*r* = 0.2954, *p* < 0.05) (Figure 4A). In contrast, Pearson’s correlation analysis revealed no statistically significant correlations between serum periostin and IL-13, eosinophil count in dogs with cAD (*r* = 0.1922, *p* = 0.17 and *r* = 0.2283, *p* = 0.15, respectively) (Figure 4B,C).

#### Correlation between periostin concentrations and other biomarkers in healthy dogs

Pearson’s correlation analysis revealed no statistically significant correlations between serum periostin and IL-13 (*n* = 19) in control group (*r* = 0.2706, *p* = 0.26) (Figure 4E). And Spearman’s correlation analysis revealed no statistically significant correlations between serum periostin and IL-4 (*n* = 19), eosinophil count (*n* = 14) in healthy dogs (r= −0.2145, *p* = 0.38 and r= −0.0083, *p* = 0.98, respectively) (Figure 4D,F).

### Pre- and post-treatment comparison of serum periostin concentrations, IL-4 and IL-13 levels, and eosinophil counts in dogs with cAD

**Figure 5.**
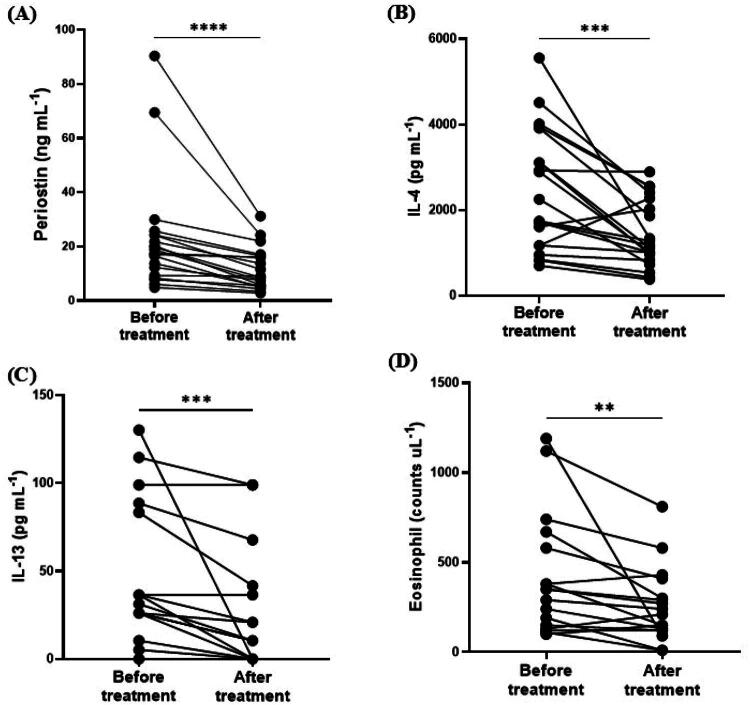
Comparison of serum periostin concentrations, IL-4 and IL-13 levels, and eosinophil counts pre- and post-treatment with prednisolone or oclacitinib in dogs with cAD. Statistical significance was determined using the Wilcoxon matched-pairs signed rank test, with **p < 0.01, ***p < 0.001, and ****p < 0.0001.

To evaluate changes in serum periostin concentrations, we retrospectively analyzed samples from 20 dogs with cAD that were available for follow-up after receiving additional treatment. All dogs were treated with either prednisolone (PDS) or oclacitinib. Compared with pre-treatment concentrations, serum periostin concentrations significantly decreased after treatment (median: 18.11 ng/mL, IQR: 8.540–24.19 vs. median: 8.789 ng/mL, IQR: 5.372–16.37; *p* < 0.001). In addition, significant reductions in serum IL-4 and IL-13 levels were observed after treatment (IL-4: median: 1,995 pg/mL, IQR: 1,171–3,715 vs. median: 1,143 pg/mL, IQR: 862.8–2,214; *p* < 0.001, IL-13: median: 26.05 pg/mL, IQR: 1.308–71.62 vs. median: 5.215 pg/mL, IQR: 0.0–32.56; *p* < 0.001). Furthermore, in the 17 dogs with available complete blood count measurements, eosinophil counts significantly decreased following treatment (median: 350/uL, IQR: 140–625 vs. median: 210/uL, IQR: 120–355; *p* < 0.01) (Figure 5).

## Discussion

Recent findings have underscored the pivotal role of periostin in the pathophysiology of AD, particularly in conjunction with cytokines such as thymic stromal lymphopoietin, IL-5, IL-13, and IL-4 (Majewska et al. 2016). These cytokines are integral to the type 2 immune response, which has been well-established as a central mechanism in the development of AD, including in canine models (Marsella 2021). While prior studies have predominantly focused on the expression of periostin within skin tissues of affected dogs, our research has extended these findings by demonstrating a marked elevation in serum periostin concentrations in dogs suffering from cAD as compared with healthy controls. This elevation is likely the result of periostin secretion at sites of inflammation and its subsequent released into the bloodstream (Kim et al. 2014; Matsusaka et al. 2015). As periostin is secreted basolaterally and vascular structures permeate mesenchymal tissues, it represents a promising candidate for a blood-based biomarker indicative of epithelial type 2 activation.

This study highlights the utility of serum periostin concentration as a potential objective marker for assessing the severity of cAD. Previous studies have examined the relationship between lesion severity scores in cAD and various biomarkers such as macrophage migration inhibitory factor, IL-17, and IL-31 levels (Chaudhary et al. 2019; Gow et al. 2019). However, consistent correlations between serum levels of these biomarkers and disease severity have been difficult to establish. In contrast, our findings demonstrate a clear and significant correlation between serum periostin concentrations and CADESI-04 scores. These results are consistent with observations in human studies, where periostin levels have been associated with the severity of AD in both children and adults (Kou et al. 2014; Sung et al. 2017). Unexpectedly, our study did not identify a significant correlation between serum periostin concentrations and PVAS scores. It has been suggested that periostin may contribute to pruritus by stimulating nerve fibers (Hashimoto et al., 2021; Hashimoto et al., 2021). However, the absence of a correlation in our findings might be explained by the subjective nature of the PVAS score, which relies on owner-reported assessments and may lack the objectivity of CADESI-04 scores. Further investigations are needed to better understand the exact role of periostin in the pathogenesis of pruritus. Despite this limitation, we propose that serum periostin represents a promising biomarker, potentially overcoming some of the subjectivity inherent in CADESI-04 scoring and offering a more reliable measure of disease severity.

AD primarily develops through the activation of Th2 cells following the transdermal entry of allergens, which subsequently leads to the release of Th2-associated cytokines such as IL-4, IL-13, and IL-5 (Grewe et al. 1998; Brandt and Sivaprasad 2011). Among these, IL-4 and IL-13 play key roles in the Th2 immune response, as they stimulate fibroblasts to produce periostin, a matricellular protein involved in tissue remodeling and inflammation (Masuoka et al. 2012; Izuhara et al. 2019). In the present study, serum IL-4 and IL-13 concentrations were significantly elevated in dogs with cAD compared to healthy controls. Previous studies in humans have demonstrated a positive correlation between periostin and Th2 cytokines, including IL-4 and IL-13, particularly in bronchial asthma and atopic dermatitis patients (Takayama et al. 2006; Masuoka et al. 2012; Jia et al. 2012). Consistent with these findings, our study also identified a significant positive correlation between serum IL-4 and periostin concentrations within the cAD group. In contrast, no statistically significant correlation was observed between IL-13 and serum periostin levels in dogs with cAD. This discrepancy may be attributed to physiological or species-specific differences between dogs and humans. Although periostin expression is known to be upregulated by Th2 cytokines, unidentified inhibitory or regulatory factors in cAD may suppress or downregulate periostin expression. Therefore, further investigation at the molecular level is needed to elucidate the regulatory mechanisms of periostin expression in dogs with cAD. Meanwhile, eosinophil counts, which are known to be regulated by IL-5 (Pelaia et al. 2019), were significantly elevated in the cAD group compared to healthy controls. However, no significant correlation was found between eosinophil counts and serum periostin concentrations in the cAD group. This finding suggests that eosinophils may not act as direct inducers of periostin, and that species-specific differences could have contributed. In healthy controls, the animals were clinically healthy at the time of sampling, and most exhibited low levels of IL-4, IL-13, and eosinophils. As a result, no clear correlation with serum periostin levels was observed in this group.

We demonstrated that serum periostin concentrations decreased in dogs treated with PDS or oclacitinib, the first-line therapies for cAD, corresponding with clinical improvement. Currently, the evaluation of therapeutic responses in dogs with cAD primarily relies on subjective assessments performed either by veterinarians evaluating clinical signs or by owners rating the severity of pruritus in their dogs. Thus, this study is particularly interesting as it provides an objective method to assess treatment responses. Furthermore, we concurrently evaluated changes in IL-4 and IL-13 levels and eosinophil counts post-treatment, which are closely associated with Th2 inflammation. Both glucocorticoids and oclacitinib are known to inhibit Th2 cells (Richards et al. 2000; Jasiecka-Mikołajczyk et al. 2021). Our study demonstrated a marked reduction in Th2-derived cytokines and eosinophils in dogs who successfully responded to treatment. These findings emphasize the importance of targeting Th2 inflammation in the management of cAD and highlight the potential of therapies designed to modulate this specific immune response pathway.

This study was subject to several limitations that should be acknowledged. First, the marked disparity in sample size between the control group and the cAD cohort may have introduced bias, potentially influencing the study outcomes. Second, the retrospective design limited the availability of blood examination data and residual samples for dogs with cAD, preventing a comprehensive analysis across all subjects such as additional measurement of pruritic cytokines (IL-31) associated with cAD. Third, despite the well-established correlation between the chronicity of AD and serum periostin concentrations in human medicine (Kou et al. 2014), this study was unable to explore this association due to data constraints. Finally, this study evaluated serum periostin concentrations in response to PDS and oclacitinib but did not assess responses to other treatments such as topical glucocorticoids, cyclosporine, and lokivetmab. To address these limitations, future research should adopt a prospective design with a larger and more balanced sample size and investigate periostin responses to a broader range of treatments to better validate its clinical utility in cAD.

To the best of our knowledge, this is the first research to investigate the relationship between serum periostin concentrations and cAD severity. This study demonstrated that serum periostin is a valuable potential biomarker for objectively assessing the severity of disease and treatment response in cAD. Our findings provide four key insights into the role of periostin in cAD. First, serum periostin concentrations were significantly higher in dogs with cAD than in healthy controls. Second, there was a positive correlation between serum periostin concentrations and disease severity. Third, serum periostin concentrations in cAD group were associated with Th-2 cytokine, IL-4 level. Finally, serum periostin concentrations decreased significantly following treatment, highlighting the potential utility of serum periostin as a dynamic biomarker for monitoring therapeutic responses. These results emphasize the potential of serum periostin as a reliable measure for monitoring disease progression and evaluating therapeutic outcomes in cAD that can provide supplementary support to subjective scoring systems commonly used in clinical practice. Future studies with larger, prospectively collected datasets are needed to validate and expand upon these results. Nonetheless, this research marks a significant advancement in the objective evaluation of cAD severity and treatment efficacy.
